# Activation of the Left Inferior Frontal Gyrus in the First 200 ms of Reading: Evidence from Magnetoencephalography (MEG)

**DOI:** 10.1371/journal.pone.0005359

**Published:** 2009-04-27

**Authors:** Piers L. Cornelissen, Morten L. Kringelbach, Andrew W. Ellis, Carol Whitney, Ian E. Holliday, Peter C. Hansen

**Affiliations:** 1 Department of Psychology, University of York, York, United Kingdom; 2 Department of Psychiatry, University of Oxford, Oxford, United Kingdom; 3 Department of Physiology, Anatomy and Genetics, University of Oxford, Oxford, United Kingdom; 4 Department of Linguistics, University of Maryland, College Park, Maryland, United States of America; 5 The Wellcome Trust Laboratory for MEG Studies, School of Life and Health Sciences, Aston University, Birmingham, United Kingdom; 6 School of Psychology, University of Birmingham, Birmingham, United Kingdom; University of Groningen, Netherlands

## Abstract

**Background:**

It is well established that the left inferior frontal gyrus plays a key role in the cerebral cortical network that supports reading and visual word recognition. Less clear is when in time this contribution begins. We used magnetoencephalography (MEG), which has both good spatial and excellent temporal resolution, to address this question.

**Methodology/Principal Findings:**

MEG data were recorded during a passive viewing paradigm, chosen to emphasize the stimulus-driven component of the cortical response, in which right-handed participants were presented words, consonant strings, and unfamiliar faces to central vision. Time-frequency analyses showed a left-lateralized inferior frontal gyrus (pars opercularis) response to words between 100–250 ms in the beta frequency band that was significantly stronger than the response to consonant strings or faces. The left inferior frontal gyrus response to words peaked at ∼130 ms. This response was significantly later in time than the left middle occipital gyrus, which peaked at ∼115 ms, but not significantly different from the peak response in the left mid fusiform gyrus, which peaked at ∼140 ms, at a location coincident with the fMRI–defined visual word form area (VWFA). Significant responses were also detected to words in other parts of the reading network, including the anterior middle temporal gyrus, the left posterior middle temporal gyrus, the angular and supramarginal gyri, and the left superior temporal gyrus.

**Conclusions/Significance:**

These findings suggest very early interactions between the vision and language domains during visual word recognition, with speech motor areas being activated at the same time as the orthographic word-form is being resolved within the fusiform gyrus. This challenges the conventional view of a temporally serial processing sequence for visual word recognition in which letter forms are initially decoded, interact with their phonological and semantic representations, and only then gain access to a speech code.

## Introduction

Like most complex behaviours, visual word recognition is thought to result from the dynamic interplay between the elements of a distributed cortical and sub-cortical network. To fully understand how visual word recognition is achieved, we need to identify the necessary and sufficient compliment of nodes that comprise this network – its functional anatomy. We also need to understand how information flows through this network over time, and indeed how the structure of the network itself may change with time during the process of recognition.

In order to chart the spatiotemporal evolution of cortical events during the first half-second of visual word recognition, Pammer et al. [1] recently employed magnetoencephalography (MEG) in combination with beamforming analyses [2]–[4]. Before describing the findings of Pammer et al. [1], we will introduce the beamforming techniques that were originally developed to improve the sensitivity of fixed array radars to locate signals of interest [5]. More recently, these algorithms have been exploited successfully to reconstruct the neuronal sources generating MEG data [2], [3], [6]–[8]. In a beamforming analysis, the neuronal signal at a location of interest in the brain is constructed as the weighted sum of the signals recorded by the MEG sensors, the sensor weights computed for each location forming a so-called “virtual electrode”. The beamformer weights are determined by an optimization algorithm so that the signal from a location of interest contributes to the beamformer output unattenuated, whereas the signal from other locations is suppressed. For a whole brain analysis, a cubic lattice of virtual electrodes is defined within the brain, and an independent set of weights is computed for each of them. The main assumption behind beamforming analysis is that the time series from distinct cortical areas are not perfectly linearly correlated (e.g. Robinson & Vrba [2]), an assumption which has found broad theoretical and empirical support [9], [10]. A major advantage of beamformer analysis relative to alternative source localisation techniques such as equivalent current dipole modelling or minimum norm estimation (which take evoked-average data as input) is the ability to image changes in cortical oscillatory power that do not give rise to a strong signal in the evoked-average response [11]. Beamforming has previously been employed in a variety of studies, including investigations of the Stroop phenomenon [12], the functions of the motor cortex [13] and the human somatosensory cortex [14]. It has been shown to be able to reveal changes in cortical synchronization that are spatially coincident with the haemodynamic response found with fMRI [15], [16]. Further discussion of beamforming techniques can be found in Ioannides [6], Salmelin [7], and Singh [8].

Participants in the Pammer et al. [1] study were shown a mixture of real words (e.g., HOUSE) and nonwords that were anagrams of real words (e.g., HOSUE), and were asked to press one of two buttons to indicate whether the stimulus was a word or not (lexical decision). By comparing the signal power during conditions in which the brain was engaged in active processing with baseline conditions, these authors quantified both increases (event-related synchronization, ERS) and decreases (event-related desynchronization, ERD) in cortical oscillations at each virtual electrode (c.f. Pfurtscheller & Lopes da Silva [17]). There are three aspects of the data from Pammer et al. [1] that are of particular relevance to the present study. The first is an ERS response observed in the posterior parts of the middle and inferior occipital gyri (BA 18), extending into the lingual gyri and cunei. This response was seen in both hemispheres, though it was more pronounced in the LH (peak at MNI *X* = −14, *Y* = −88, *Z* = −6). The response was present in the 0–200 and 100–300 ms active windows, but was absent from later time windows. That could mean that the middle occipital gyrus (MOG) response is short-lived or that it is stimulus-bound and only occurs when a stimulus is visible on the screen (which was for the first 200 ms). Similar activations were reported in MEG studies by Tarkiainen et al. [18], Salmelin et al. [19] and Cornelissen et al. [20] using equivalent current dipole modelling, by Dhond et al. [21] and Marinkovic et al. [22] using minimum norm current estimation, and by Kujala et al. [23] using dynamic imaging of coherent sources (DICS). Like the equivalent response in fMRI studies [24], this posterior MEG response has usually been associated with the encoding of letter shapes in words and other alphabetic strings.

Pammer et al. [1] also observed an ERD response to words and anagrams in the fusiform gyri that was stronger in the left fusiform gyrus than the right. The response was first visible in the 100–300 ms active window then expanded in later time windows in both the posterior–anterior and medial–lateral directions to include more anterior parts of the inferior left temporal lobe. The peak for this activation was MNI *X* = −32, *Y* = −64, *Z* = −6, which is close to, but somewhat more medial and more posterior than the fMRI peak reported for the so-called ‘visual word form area’ (VWFA: MNI *X* = −44, *Y* = −58, *Z* = −15: Jobard et al., [25]). The implied time course of the left mid FG response was in good agreement with the timing of word-specific responses from event-related potentials [26] and intracranial field potentials [27] which suggest an activation that peaks at 180–200 ms. In the Pammer et al. [1] data a similar response was observed for anagram stimuli, though it appeared to be delayed by around 50 ms. Most fMRI studies find similar levels of activation to real words and legal nonwords (‘pseudowords’) at the putative VWFA [25], [28], implying that the role of the VWFA may be to formulate abstract perceptual descriptions of words and potential words that are independent of factors such as where the stimulus appears in space, and the physical form in which it appears (case, font, etc; McCandliss et al., [29]). The forward spread of activation into anterior temporal regions seen in the results of Pammer et al. [1] could plausibly be associated with the activation of semantic representations [27], [30]–[32]. This could explain why the ERD response for anagrams did not extend as far anteriorly into the temporal lobes as did the response to familiar words (see Figure 2 of Pammer et al., [1]).

The third and final feature of Pammer et al.'s [1] results which is relevant to the present study is the response they observed in the posterior superior part of the inferior frontal gyrus (IFG), particularly within the *pars opercularis*, extending into the precentral gyrus (BA44/6). That response showed a peak at MNI *X* = −60, *Y* = 8, *Z* = 22. It was seen in the first active window (0–200 ms) and then spread inferiorly and anteriorly over time. Wilson et al. [33] also reported left IFG (*pars opercularis*) responses to written words and legal nonwords but not consonant strings in an MEG study using equivalent current dipole analysis. The left IFG responses in that study preceded other perisylvian responses (in the superior temporal sulcus, superior temporal gyrus and supramarginal gyrus), and were faster to words than to pseudowords. Pammer et al. [1] noted that the left IFG activation in their study fitted with the cortical regions associated with phonological speech processing in the meta-analysis by Bookheimer [34], partly on the basis of its involvement in silent reading and naming [35]–[37] where some studies have reported stronger activation to low frequency words and nonwords than to high frequency words [35], [38]. Heim et al. [39] proposed that this area lies at the phonological end of nonlexical grapheme-phoneme conversion processes in reading.

### The Current Study

The findings of the Pammer et al. [1] study were preliminary. Important obstacles to interpretation included the fact that only visual words were used, so the potential for IFG to respond to non-alphabetic stimuli could not be ascertained (see e.g. Halgren et al. [40] for evidence of rapid responses to faces in IFG). In addition, subjects were asked to respond with their right hand and to withhold responses until cued. Therefore, it was conceivable that the early activity in left IFG could be related to motor preparatory processes and not visual word recognition per se. For example, Fink et al. [41] point out that the pars opercularis of the IFG has been shown to be activated during the observation and recognition of actions performed by others [42] and in the observation and subsequent imitation of actions [43]. These findings have led to the suggestion that the ventral premotor cortex, together with the pars opercularis of the IFG in humans might be part of the “mirror neurone system” [43]. Finally, the pars opercularis of the IFG has even been suggested to play a role in local visual search tasks in experiments where participants are asked to judge whether a simple figure is embedded in a more complex figure, as compared with judging whether a simple figure matches a highlighted portion of a more complex figure [41]. Therefore, the main aim of the current study was to better characterize early activation of IFG - specifically in the pars opercularis and precentral gyrus - in the brain's response to visually presented words. The experiment to be reported involved passive viewing of blocks of words, consonant strings and unfamiliar faces in order to emphasize the stimulus driven, automatic components of cortical processing. We chose a pseudorandomized blocked design to minimize the dynamic effects related to changing task set from one trial to the next and to ensure that participants were in a relatively stable mode of processing for each stimulus class. The explicit task for the (right-handed) participants was to monitor a small red fixation cross in the centre of a projection screen, pressing a button with their *left* hand if it changed from red to green on infrequent catch trials. On a given experimental trial, the central fixation cross was replaced for 300 ms by a common 5-letter word, a consonant string, or an unfamiliar face. The primary question at issue was whether the word stimuli would evoke an early response from the left IFG, and how the strength and time course of that response would compare with the response to consonant strings and unfamiliar faces. A stronger left IFG response to words than consonant strings would be consistent with activation of language processes beyond orthography, such as phonological or semantic processing. However, an equally strong response to unfamiliar faces might imply that the left IFG was less concerned with phonological/semantic processing and more concerned with, for example, the control of attentional resources in a situation where participants had to monitor the colour of the fixation cross while ignoring irrelevant but attention-grabbing stimuli (words, consonant strings and faces). Suggestions such as these for a role for left IFG in cognitive control have been proposed by, amongst others, Snyder et al. [44]. Therefore, it is important to exclude this more general role for IFG in visual word recognition and reading, before focusing research exclusively on more specific possibilities such as phonological and/or semantic processing.

Data analysis concentrated on the first 500 ms after presentation of a stimulus. Beamformer maps were generated using the same length of passive and active windows (i.e. 200 ms) as well as the same frequency band (10–20 Hz) as Pammer et al. [1] to allow a comparison of our results with theirs, especially with respect to the evolving neural responses to words. But moving time windows provide only partial insights into the time course of processing. A response may be detected in a particular brain region within the first 200 ms, but one does not know whether the response was present from the start (e.g., an anticipatory response triggered by the presentation of the fixation cross) or was a reaction to the presentation of the stimulus. If the latter, the moving time windows approach provides only a first guide to the time course of the response, and certainly fails to take full advantage of the potential of MEG for millisecond timing of events. Much better temporal resolution can be obtained by identifying regions of interest (ROIs) based on the whole brain analyses, then reconstructing virtual electrode outputs targeted specifically at each ROI. This selective virtual electrode output can be analyzed and interpreted in different ways.

We will present two forms of analysis. The first are *time-frequency plots* which show how the power of the response of a virtual electrode varies over time at different frequencies (cf. Maratos et al., [45]). We will use such plots to compare the left IFG's response to words with its response to consonant strings and faces; also to compare the left IFG's response to words with the responses of three comparison sites (the right IFG, the left MOG and the left mid FG / VWFA). The total power in a time-frequency plot can be broken down into two components [45]. *Evoked components* are attributable to those responses which have a stereotypical wave shape that is sufficiently phase-locked to the onset of a stimulus to be revealed both by the evoked average in the time domain and by analysis in the frequency domain. *Induced components* are those changes in oscillatory activity which, though they may occur within a predictable time-window following stimulus onset, lack sufficient phase locking to be revealed by averaging in the time domain. They are however revealed by changes in power in the frequency domain [11], [46].

The second type of virtual electrode analysis that will be presented here focuses on evoked responses in the time domain. The result is a single time series for a given condition of the experiment at a given location in the brain that shows changes in the amplitude of the neural response with millisecond accuracy. *Event related field (ERF)* plots of this type resemble the event-related potentials (ERPs) of EEG studies but arise from selected points of interest within the brain. We will present ERF plots showing how the strength of the evoked response to words at the left IFG, left MOG and left mid FG (VWFA) changes over time. The peak of the ERF response will be treated as a marker for the relative timing of the responses to words at those three locations which constrains theorizing regarding the likely flow of information between the three areas - a strategy which has been reported by a number of other researchers (e.g. Dhond et al. [47]; Salmelin et al. [19]; Tarkiainen et al. [18]). Are the data compatible, for example, with a temporally linear processing sequence in which letter forms or features are first analyzed at the left MOG, with processing progressing down the left fusiform gyrus, resulting in the creation of an abstract orthographic representation at the VWFA, and with abstract orthographic representations then being used to compute phonological representations at the left IFG? Or do the data fit better with a parallel temporal processing account in which the left MOG provides input to more abstract orthographic processing along the left fusiform gyrus while *at the same time* activating the left IFG?

## Results

### Whole Brain Analysis of Word, Consonant String, and Face Responses at 10–20 Hz

**Figure 1:**
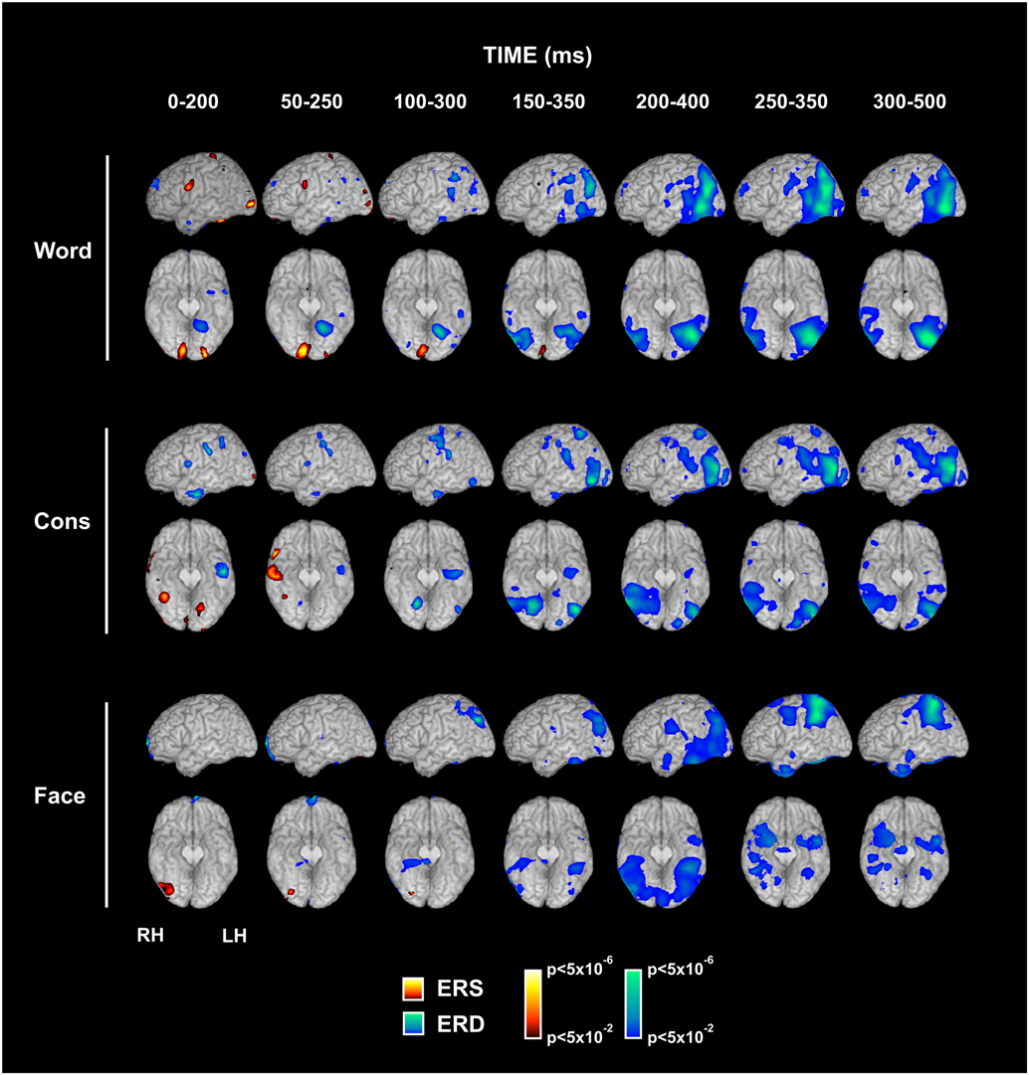
Temporal evolution of left hemisphere and ventral brain activity elicited by written words, consonant string, and faces. The figure shows the beamformer group analysis of brain activity in the beta frequency band for successive 200 ms long windows of interest, each separated in time by 50 ms, and superimposed on a canonical brain with the cerebellum removed.

Figure 1 shows the results obtained by beamformer analysis in the 10–20 Hz frequency band (i.e. replicating the beamformer parameters used by Pammer et al. [1]) for the words, consonant strings and unfamiliar faces conditions for comparisons of moving active time windows with a constant passive window of −200 to 0 ms. Increases in power (ERS) are shown in red-yellow while decreases in power (ERD) are shown in blue.

Consistent with Pammer et al. [1], Figure 1 shows a bilateral, posterior increase in power (ERS) in the words condition affecting the MOG in each hemisphere (BA 18/19) and extending into the lingual gyrus and cuneus. This response is present in the 0–200 ms time window, remains visible through to the 150–350 ms time window, but has disappeared by 200–400 ms. The response is not visible in the consonant strings condition or the faces condition which shows an ERS response in the right inferior occipital cortex.

In the words condition, a decrease in power (ERD) appears in the first time window (0–200 ms) in the left mid FG. That response extends over time in both medial-to-lateral and posterior-to-anterior directions. A right mid fusiform response is visible in the later time windows but remains weaker than the response in the left mid FG. The nature and timing of this response to words is similar to that observed by Pammer et al. [1]. The left mid fusiform response is smaller and much delayed in the consonant strings condition. In the faces condition, the earliest fusiform activation occurs in the right hemisphere close to the site of the ‘fusiform face area’ [48], [49], extending to the left hemisphere in later time windows.

Importantly, the words condition shows a clear, early response in the left dorsal IFG (pars opercularis, BA 44/6) in the form of an increase in power (ERS) in the 0–200 and 50–250 ms active windows. That response disappears by the 100–300 ms window. Consonant strings show a weaker response (decrease in power; ERD) in the first two early time windows. There is no significant early left IFG response in the faces condition. For words and, to a lesser extent, consonant strings, later time windows also show activation in other parts of the reading network, including the anterior middle temporal gyrus (BA 21 and 38), the left posterior middle temporal gyrus (BA37/39), the angular and supramarginal gyri (BA 39/40), and the left superior temporal gyrus (BA 22).

### Time-Frequency Analysis of the Response of the Left IFG to Words, Consonant Strings, and Faces

Time-frequency analyses were carried out using the FieldTrip toolbox developed at the F. C. Donders Centre for Cognitive Neuroimaging (http://www.ru.nl/fcdonders/fieldtrip) using Matlab 7.0.4 (MathWorks, Natick, MA). Time-frequency plots for the period 0–500 ms were calculated using a Morlet wavelet transform in the frequency range 5–40 Hz.

**Figure 2:**
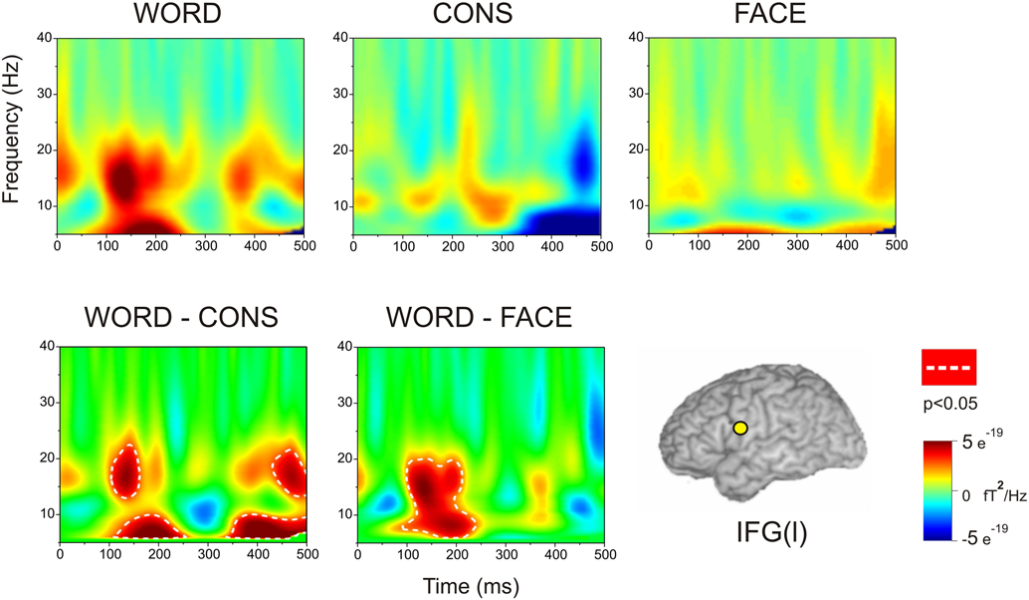
The upper row shows the time-frequency plots for words, consonant strings, and faces for the left IFG ROI. The lower row shows the differences between the time-frequency plots comparing words with consonants strings and words with faces. The white dotted lines represent regions in the time-frequency plots within which the difference between conditions reached significance at p<0.05, according to the general linear mixed models.

The upper row of Figure 2 shows grand average time-frequency plots for the left IFG response to words, consonant strings and faces. Amplitude changes per time frequency bin were computed relative to a −250–0 ms baseline. Increases in power relative to the baseline are shown in yellow-red while decreases are shown in blue. The pale green background shows the regions in the time-frequency plot where power levels were comparable in the active and passive (baseline) periods. A left IFG response to words is visible between ∼100 and ∼250 ms in the 0–25 Hz frequency band that is strongest in the 10–25 Hz band between 100 and ∼250 ms. The responses to consonant strings (top row, middle) and faces (top row, right) lack the strong early response shown by words.

The two images at the bottom of Figure 2 show the results of statistical comparisons between the time-frequency response of the left IFG to words and its responses to consonant strings and faces. These comparisons were made using PROC MIXED in SAS (SAS Institute Inc., North Carolina, US) to compute a generalized linear mixed model (GLMM). Time-frequency plots were treated as two-dimensional arrays of small time-frequency tiles. As in any other spatial analysis, the values of the observed variables at each location in the resulting 2-D array cannot be assumed to be statistically independent. In the present data, the power at any single time-frequency tile will typically covary with the power in other tiles in inverse proportion to the distance between tiles in the 2-D array. Therefore in the GLMM comparing the different stimulus conditions (words *vs.* consonant strings, or words *vs.* faces) a repeated measures factor was included to account for the fact that each participant's time-frequency plot is made up of multiple time-frequency tiles. We also controlled for time-frequency (or spatial) co-variance in the spectrogram by assuming the estimates of power followed a Gaussian distribution. Consequently a Gaussian link function was used in the model for the outcome. The time-frequency (spatial) variability was integrated into the model by specifying an exponential spatial correlation model for the model residuals.

Dotted white lines in the lower images in Figure 2 enclose regions where there were significant differences in power (*p*<0.05) at the left IFG for words compared with consonant strings and faces. These statistical contours are based on the estimated marginal means derived from the model parameters and these predicted population margins were compared using tests for simple effects by partitioning the interaction effects. These analyses confirm the impression created by comparing words with consonant strings and faces in the upper row of Figure 2: words generated significantly stronger left IFG responses than consonant strings or faces from ∼100 to ∼250 ms in the 5–25 Hz region. The comparison between words and consonant strings showed an additional significant difference from ∼350 to ∼500 ms in the 5–25 Hz region which in part reflected the loss of power in the later response to consonant strings relative to the baseline period.

### Total, Evoked, and Induced Responses to Words in the Left IFG, Right IFG, Left MOG, and Left Mid FG (VWFA)

**Figure 3:**
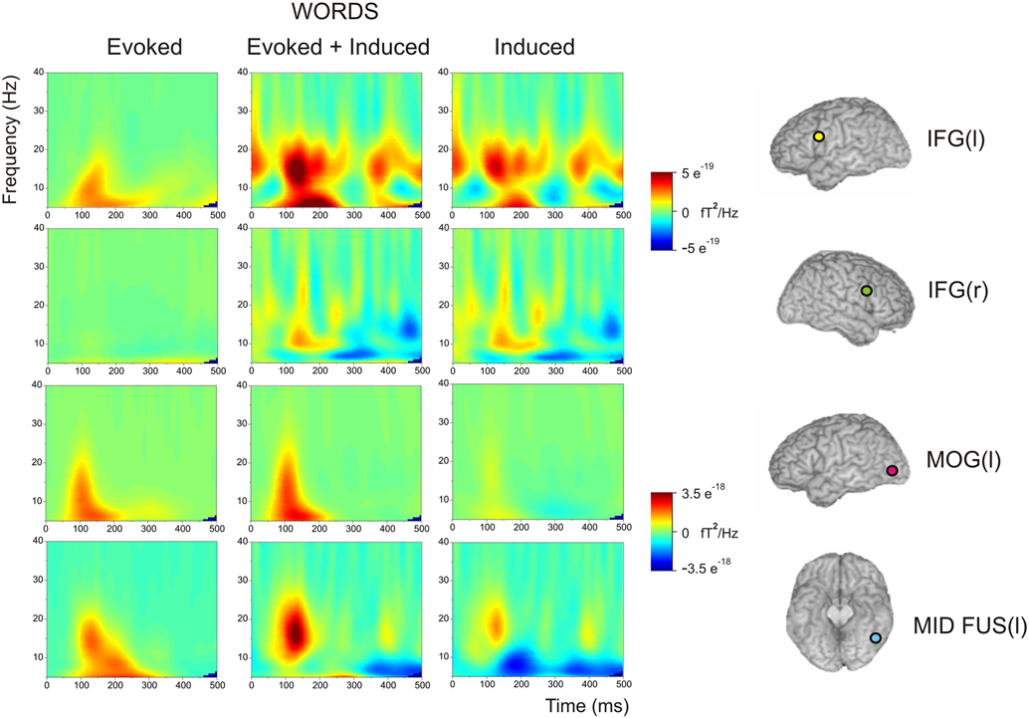
Shows time frequency plots for all four ROIs for all participants' responses to words. The left column represents evoked activity, the middle column presents evoked plus induced activity, while the right column represents induced activity alone.

As noted in the Introduction, frequency domain analyses of MEG time series data conventionally distinguish evoked from induced components. The image at the top centre in Figure 3 is the same as the image at the top left of Figure 2, showing the total (evoked+induced) response of the left IFG to words in the time-frequency domain. The image at the left of the top row in Figure 3 shows the evoked component of the response words produced. The image at the right of the top row shows the induced component of the left IFG response to words, which is generated by simply subtracting the evoked response from the total response. The evoked response of the left IFG to words, which is predominantly in the 0–20 Hz range, starts between 50 and 100 ms, peaks between 100 and 150 ms, and has faded away by 300 ms. The induced response to words is strongest in the 10–25 Hz frequency band and appears to be periodic in character, but with a peak value at ∼120 ms.

Figure 3 also shows the total, evoked and induced responses to words for the right IFG, left MOG and left mid FG (VWFA) sites. The right IFG shows a much weaker response to words than in the left IFG, which is mostly in the induced component. The comparison of the left and right IFG responses shows that the IFG response to words is quite strongly lateralised to the left hemisphere. The left MOG, which appeared to have an early ERS response in the beamformer maps (Figure 1), displays a strong total response in the 0–25 Hz range. This appears to arise mostly from the evoked component. It has a similar time course to the left IFG response but appears to peak a little earlier. The left mid FG (VWFA) response has a stronger evoked component than an induced component, lies mostly in the 0–20 Hz range, and has a similar time course to the left IFG.

### Time Domain (Event Related Fields) Analysis of Responses to Words in the Left IFG, Left MOG, and Left Mid FG (VWFA)

In the final set of analyses, the three left hemisphere ROIs were analysed in the time domain rather than the frequency domain. These analyses only reflect the evoked component of the response, so the right IFG, which showed little or no evoked response to words, was excluded at this stage. These analyses were only applied to the data from the words condition of the experiment on the grounds that the left IFG showed diminished response to consonant strings and no response to unfamiliar faces (see Figure 2). The purpose of these analyses was to obtain further evidence for the temporal sequence of events in left MOG, left IFG and left mid FG (VWFA).

**Figure 4:**
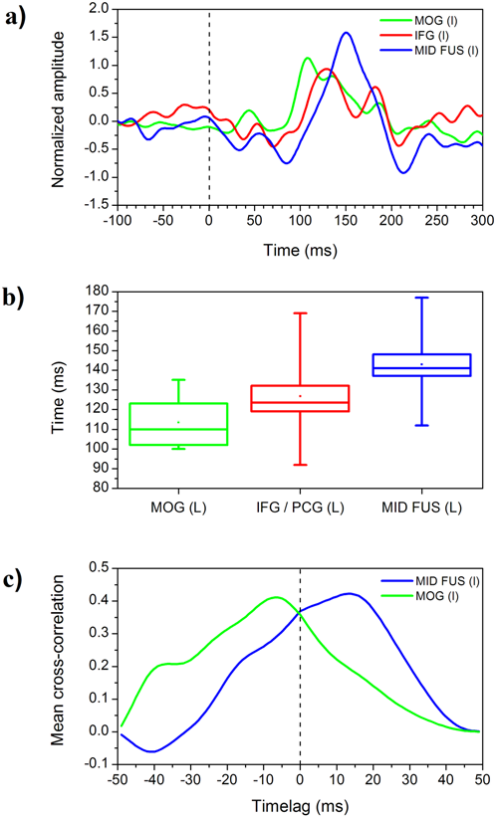
Normalised ERFs, peak latency, and mean cross-correlation. (A) Shows the normalised ERFs for centrally presented words in the left MOG (green), left IFG (red), and left MID FUS (VWFA) in blue. (B) Shows box and whisker plots for the mean peak latency in the same ROIs. (C) Shows the mean cross-correlation between IFG and left MID FUS (VWFA) in blue and IFG and left MOG (green).

Event-related fields (ERFs) were computed for each location by low-pass filtering the time series at 40 Hz (cf. Tarkiainen et al., [50]). The time series were averaged for each condition and each participant from 200 ms before stimulus presentation to 700 ms after stimulus onset, and amplitudes normalised across conditions for each participant. The upper part of Figure 4 shows the resulting mean ERF plots across participants for the words condition at the left IFG, left MOG and left mid FG (VWFA) from −100 ms to 300 ms post onset (by which time the evoked responses have largely passed). Consistent with Figure 3, the evoked response to words at each site begins between 50 and 100 ms, peaks between 100 and 150 ms, and is finished by around 200 ms. There is clearly overlap between the time courses of the evoked response at each site.

The latencies of the peak responses at each location were extracted from each participant's time series using a 9-point, 2nd order Savitsky-Golay filter. This method provides greater precision for locating peaks, compared to simple band-pass filtering [51]. The lower part of Figure 4 presents box-and-whisker plots of the mean latencies of the peak responses to words at each virtual electrode site. The lower and upper horizontal lines of each plot show the fastest and slowest peak latencies at each location. Each box shows the middle 50% of peak latencies. The horizontal line within a box shows the median peak latency while the dot towards the centre shows the mean peak latency. The evoked response of the left IFG peaked at a mean of 127 ms (sd 19 ms) post stimulus onset, with a range from 92 to 169 ms. The mean peak latency for the left MOG occurred somewhat earlier at 113 ms (sd 12 ms). The range of peak latencies for the left MOG (from 100 to 135 ms) is more restricted than the range for the left IFG. The mean peak latency for the left mid FG (VWFA) was slowest at 143 ms (sd 17 ms), with a range from 112 to 177 ms. The significance of the differences between the peak latencies of the evoked responses to words at the three sites was determined using a one factor (i.e. virtual electrode site), repeated measures mixed model using PROC MIXED in SAS (SAS Institute Inc., North Carolina, US) to compare the latencies of the peaks in the words condition extracted from each participant at each location. The main effect of virtual electrode site was highly significant, *F*(2,9) = 22.7, *p*<0.001. Planned comparisons of the least square mean differences in peak latencies showed that the peak latency of the left MOG response occurred significantly earlier than the peak latencies at both the left IFG, *t* = −2.94, *p*<0.05, and the left mid FG (VWFA), *t* = 4.65, *p*<0.01. The difference in latencies between the left IFG and the left mid FG (VWFA) was not significant (due in part to the greater variability in peak latencies at those two sites).

These conclusions about the relative timing of activation in different ROIs are based on group-averaged timecourses. However it is known that estimates of peak activation can be uncertain both between electrode sites at the group level, and within electrodes sites across trials [52]. Therefore, to provide a converging line of evidence for the relative timings between our three ROIs, we ran a further analysis in which we computed the mean cross-correlation function between IFG and the other two ROIs for centrally presented words. As is shown in Figure 4C), over the 200 ms window for which cross-correlations were computed, IFG shows a phase advance of ∼20 ms compared to VWFA, and a phase lag of ∼10–15 ms compared to left and right MOG.

## Discussion

The main focus of the present study was on the response to written words in the left dorsal IFG (*pars opercularis*). Could we provide further evidence of the rapid response shown in the results of Pammer et al. [1]? How would the left IFG's response to words compare with its response to consonant strings and to faces? How would it compare with the responses shown by other regions of interest, notably the right IFG, left MOG and left mid FG (VWFA)? What would the time course of the response to words be in those different areas, and what might those time courses imply about their possible patterns of interaction within the larger reading network?

To facilitate a direct comparison between the present results and those of Pammer et al. [1], beamformer maps were generated showing the power at 10–20 Hz in the responses to words, consonant strings and faces in moving 200 ms active windows compared with a passive window of −200 to 0 ms. Broadly speaking, the beamformer maps for the words condition in the present study (Figure 1) represent a good match to the corresponding maps obtained by Pammer et al. [1]. Figure 1 shows an early increase in power (ERS) to words in the posterior occipital cortex, centred on the middle occipital gyri, as was the case for Pammer et al. [1]. This also corresponds to responses observed in MEG studies by Tarkiainen et al. [18], Salmelin et al. [19], Cornelissen et al. [20], Dhond et al. [21], and Kujala et al. [23] using a variety of source localisation methodologies. The left MOG response is generally taken to reflect relatively early and relatively retinotopic, analysis of letter features and letter forms.

We found a decrease in power (ERD) in the left mid fusiform gyrus (FG) that is visible in the first time window (0–200 ms; Figure 1) and which extends both laterally and anteriorly in the later time windows. The right mid FG shows a weaker response from 150–350 ms onwards. The pattern for the evolving mid FG responses is also as reported by Pammer et al. [1]. When MEG responses in the 15–25 Hz frequency band were aggregated over 500 ms and compared with a 500 ms passive window, the spatial peak of the left mid fusiform response (MNI *X* = −46, *Y* = −56, *Z* = −18) was remarkably close to the standard location of the fMRI-defined VWFA (MNI *X* = −44, *Y* = −58, *Z* = −15; Jobard et al., [25]). fMRI is based on BOLD responses gathered over periods of 5–10 seconds: it may be that the closest match between spatial peaks in fMRI and MEG will be found when the MEG responses are also aggregated over relatively long time periods. An important corollary to this is that some transient responses visible in MEG may be poorly reflected in the BOLD changes in fMRI, and therefore we should treat comparisons of results of experiments from the two imaging modalities with caution.

Figure 1 also shows an increase in power (ERS) to words in the left posterior superior IFG in the first time window. When neural activity in response to words was aggregated over the longer time period of 0–500 ms in the 15–25 Hz frequency band, a spatial peak was identified at MNI *X* = −54, *Y* = 8, *Z* = 24 (Table 1), which falls squarely within Broca's area in the *pars opercularis* of the left IFG (BA 44). In Pammer et al. [1], an early left IFG response to words was also visible, though it took the form of a decrease in power (ERD) and only became significant in the 100–300 ms active window. There are a number of procedural differences between the present experiment and that of Pammer et al. [1] which could potentially account for differences in the observed results. These include the use of passive viewing in the present experiment versus active lexical decision in the earlier study, and the fact that words in the Pammer et al. [1] study were presented for just 100 ms and were followed by a 100 ms pattern mask while words in the present experiment were presented for 300 ms and were unmasked. It is possible that the shift from an ERD to an ERS may be due to the extent to which the left IFG responses to words in the two studies were tightly time- and phase-locked to the onset of the stimuli, but further research is required to elucidate the mechanisms underlying such differences. Figure 3 shows a strong left IFG response to words that appeared partly in the induced component and partly in the evoked component. There were elements of the early response in both components, but the evoked element was strongest from 100–200 ms while the induced response was present from 0 to 500 ms. For the consonant string condition, Figure 1 indicates a significant, but smaller, response in the 0–200 ms time window in the left IFG. It should be noted however that this response took the form of a decrease in power (ERD). There was no significant response to faces in this time window in the left IFG.

**Table 1:** Listing of all peaks in the beta frequency band group analysis of the words condition, using extended 0–500 ms time windows, where t>2.5.

		MNI				
Brain Region	Hemisphere	BA	X	Y	Z	t stat
Superior Frontal Gyrus	L	6	−10	18	54	5.8
	R	6	6	26	60	5.7
**Inferior Frontal Gyrus, pars triangularis**	**L**	**45**	**−50**	**26**	**8**	**5.6**
Precuneus Cortex	R	7	2	−62	48	5.6
Intracalcarine Cortex	L	18	−6	−74	16	5.5
Inferior Frontal Gyrus, pars opercularis	L	44	−54	8	24	5.2
Occipital Pole	R	19	16	−96	10	5.1
	R	19	28	−92	26	4.4
	L	18	−32	−94	−16	3.6
Posterior Cingulate Gyrus	L	63	−4	−38	36	5.0
Lingual Gyrus	R	17	8	−90	−6	5.0
Middle Frontal Gyrus	L	6	−38	6	58	5.0
Frontal Pole	R	9	26	46	36	5.0
Paracingulate Gyrus	R	10	4	54	6	4.9
Middle Temporal Gyrus	L	21	−50	−38	−2	4.3
Superior Temporal Gyrus	L	41	−44	−32	8	4.3
Temporal Pole	R	38	52	10	−24	4.1
**Middle Occipital Gyrus**	**L**	**18/19**	**−36**	**−84**	**−2**	**3.2**
	R	18/19	44	−90	4	3.9
Lateral Occipital Cortex	R	19	44	−84	−10	3.7
Medial Orbitofrontal Cortex	R	11	10	48	−28	3.6
Supramarginal Gyrus	R	40	56	−42	22	3.5
	R	40	40	−46	44	2.9
Precentral Gyrus	R	6	42	−16	66	3.2
Lateral Occipital Cortex	R	7	32	−62	64	2.8
**Fusiform cortex**	**L**	**37**	**−46**	**−56**	**−18**	**2.7**

The peaks in bold are those that coincided with the ROI sites. BA = Brodman area.

It is worth noting that a number of other studies have also identified an early involvement of left IFG in visual word recognition. For example, in their analysis of evoked responses in a reading task measured with MEG, Salmelin et al. [19] report an early left frontal activation consistent with IFG (between 100–200 ms post-stimulus) in 5/10 stutterers and 5/10 controls. Kober et al. [53] used MEG to identify responses in Broca's and Wernicke's areas in patients who carried out a silent reading task. While Kober et al.'s [53] report focuses attention on the response in Broca's area at 720 ms post-stimulus, nevertheless an earlier peak is clear in their data at around 170 ms post-stimulus. Finally, Lachaux et al. [54] measured cortical activity from surface electrodes implanted in epilepsy patients including left IFG (*pars opercularis*). Subjects were presented two interleaved stories in a rapid serial visual presentation (RSVP) format. Words from the story to be attended to appeared in one colour, while words from the story to be ignored appeared in a different colour. Time-frequency analysis based on data averaged in relation to word onset showed clear, early beta frequency band activity for both story-lines.

In the ERP literature, a number of studies have been carried out which indicate that interactions between visual and linguistic factors during visual word recognition do begin early. For example Assadollahi and Pulvermüller [55] showed an interaction between word length and frequency in MEG, with short words exhibiting a frequency effect around 150 ms but long words at around 240 ms. Effects of lexicality (i.e. a differential response between words and pseudowords) have been reported as early as 110 ms [56], though more commonly around 200 ms [57], [58]. Lexico-semantic variables have been found to influence brain responses as early as 160 ms after visual word onset [59], [60] as has semantic coherence, a “… measure that quantifies the degree to which words sharing a root morpheme, (e.g., gold, golden, goldsmith) are related to each other in meaning” [61]. Intriguingly, Figures 5 and 7 in Hauk et al. [61] suggest early left frontal involvement particularly for semantic coherence, but unfortunately it is not possible to be more anatomically precise from their data.

In the current study, the time-frequency plots in Figure 2 show a significantly stronger response to words than to either consonant strings or faces at the left IFG between 100 and 250 ms. When that total response is broken down into evoked and induced elements (Figure 3), the early IFG response to words is reflected in both components. In comparison, the right IFG showed only a weak response that was predominantly induced. Taken together, these results show that the early left IFG response is strongly lateralised to the left hemisphere and is strongly word-specific. Pammer et al. [1] obtained an early left IFG response to words and to anagrams of real words (e.g., HOSUE, derived from HOUSE), while Wilson et al. [33] reported comparable activation of the left IFG by words and pseudowords, but less activation by consonant strings. An fMRI study by Bodke et al. [62] found activation of dorsal left IFG by words and pronounceable nonwords (‘pseudowords’) but not consonant strings or false fonts. In summary, it would therefore seem more accurate to conclude that the left IFG shows a rapid response to all word-like stimuli including to pseudowords and consonant strings.

Comparison of the peak latencies of left IFG responses with those of left MOG and left mid FG (VWFA), as shown in Figure 3 and Figure 4, indicates a substantial overlap in the time course of the evoked responses to words in these three regions. Statistical analysis of the ERFs showed, however, that the evoked response of the left MOG (mean = 113 ms) occurred significantly earlier than the evoked responses in both the left IFG (mean = 127 ms) and the left mid FG (mean = 143 ms). Numerically, the difference between the timing of the left IFG and mid FG (16 ms) was as great as the difference between the MOG and the IFG (14 ms), but the greater inter-subject variance at the mid FG and IFG meant that the difference was not significant. Importantly, however, the trend was in the direction of faster responses at the IFG than the mid FG. Pammer et al. [1] also found that the left IFG response to words was, if anything, faster than the mid fusiform response. The fact that the left IFG responds at least as quickly as the left mid FG, and 14 ms after the MOG, would appear to rule out a simple linear temporal processing account in which an initial response to letter features and forms in the left MOG leads to the creation of progressively more abstract orthographic representations in the left fusiform gyrus which, in turn, are then used to activate phonological representations at the *pars opercularis* of the left IFG.

If that linear processing account is excluded, what might be the relationship of the left IFG to the MOG and mid FG? The left MOG, mid FG and IFG are three of the nodes of a larger network responsible for reading and other aspects of language processing. We suggest that in reading, the left MOG (BA 18/19) detects letters and letter features embedded in word-like forms at around 115 ms. Activation of the MOG triggers two parallel responses. One response involves the direct activation of the left IFG (*pars opercularis*) by the MOG at around 125 ms. Dejerine [63] proposed the existence of direct anatomical connections between extrastriate visual cortex (Brodmann's areas 18/19) and lateral frontal association areas such as the IFG. DTI and histological studies of the human brains (e.g. Bürgel et al. [64]; Makris et al. [65], 2005; Wakana et al. [66]) have confirmed the presence of fibres within the superior longitudinal fasciculus (SLF) that connect the MOG to the IFG. We found a difference in latency between the left MOG (i.e. BA18/19) and the IFG of the order of only 14 ms. Assuming no additional synaptic delays, this latency difference is consistent with the conduction velocity of a myelinated fibre of about 1 μ diameter over a distance of 8–10 cm [67].

What might the function of such a fast dorsal route from MOG to IFG be? One possibility is that the same stereotypical response to any equivalent length string of letter-like objects would always be produced, irrespective of task demands i.e. whether explicit naming is required or whether stimuli are viewed passively as in the current experiment. If so, this might suggest that for a skilled reader who has had many thousands of hours of experience with print, the very presence of word-like stimuli in the visual field can trigger a response in IFG, and its role is to prime the rest of the language system to prepare for upcoming crossmodal interactions between the vision and language systems - a stimulus driven anticipatory response. It is also possible that such an effect, if true, may have been further enhanced by the blocked design of the current study. This proposal is similar to recent claims by Bar et al. [68], who showed that low spatial frequencies can facilitate visual object recognition by initiating top-down processes projected from orbitofrontal to visual cortex; object recognition elicited differential activity that developed in the left orbitofrontal cortex 50 ms earlier than it did in recognition-related areas in the temporal cortex. Bar et al. [68] suggest that these visual signals travel along the dorsal visual pathway [69], [70] the majority of whose input is derived from magnocellular layers of the lateral geniculate nucleus [71], [72]. Therefore, in this context it is interesting to note a number of studies showing predictive relationships between visual sensitivity in behavioural tasks which are known to require input from M-cells (e.g. coherent motion detection) and reading skills in school age children and adults (e.g. Cornelissen et al. [73]; Talcott et al. [74]; Sperling et al. [75]; Ben-Shachar et al. [76]), as well as differences in performance in such tasks between individuals with developmental dyslexia and normally reading age-matched controls [77], [78].

An alternative possibility is that early IFG activation in response to visually presented words reflects grapheme-to-phoneme conversion processes, perhaps along the sublexical route for reading [79], [80]. This interpretation is in line with other imaging studies that have implicated this frontal area in phonological processing during visual word recognition [81], [82], and with priming studies showing early activation of phonological representations [83]–[85]. The idea that such grapheme-phoneme conversion may involve an articulatory phonological representation is supported by recent ERP data: Ashby et al. [86] asked participants to read silently target words with voiced and unvoiced final consonants (e.g., fad and fat), preceded by nonword primes that were incongruent or congruent in voicing and vowel duration (e.g., fap or faz). Ashby et al. [86] showed that phonological feature congruency between primes and targets modulated ERP amplitudes in left frontal sensors by 80 ms post target onset.

In conclusion, the main findings from this study replicated the finding of early posterior superior left IFG / precentral activation in response to words and word-like stimuli, and is consistent with data suggesting very earlier interactions between the vision and language domains for reading. Further research is required to determine whether this represents a non-specific stimulus driven anticipatory response, or whether it is an integral part of grapheme-phoneme encoding during visual word recognition.

## Methods

### Participants and Stimuli

Ten right handed participants (6 males, 4 females) who were skilled readers with no recorded history of dyslexia or colour blindness were instructed to maintain fixation on a small red fixation cross and to press a button whenever the fixation cross changed colour to green. All participants gave written consent to take part in the study which conformed with The Code of Ethics of the World Medical Association (Declaration of Helsinki), printed in the British Medical Journal (18 July 1964). Ethical permission for the experimental procedure was provided by the Aston University Human Sciences Ethical Committee.

Three types of stimulus were presented: The word stimuli had the same characteristics as those employed by Pammer et al. [1], being familiar 5-letter, monomorphemic nouns and verbs, of medium to high Kucera-Francis frequency (mean = 168, sd = 241, range = 42–1815). Examples are COURT, FRAME and TRACE. Consonant strings were also 5 letters in length, and were unpronounceable and orthographically illegal (e.g., PFKTS). Words and consonant strings were presented in upper case letters. Unfamiliar faces were full face images, half male, half female, and cropped to obscure the hairline. All stimuli were rendered as gray-level images and were presented on a neutral gray background.

### Task and Procedure

The different experimental stimuli of each type were presented in blocks, with 64 presentations per block. The order of presentation of the different blocks was randomised across participants. Each epoch (trial) began with the presentation for 500 ms of a central, red fixation cross subtending an angle of approximately 0.2°. A word, consonant string or face was then presented centrally for 300 ms. Words and consonant strings subtended approximately 4.5° horizontally and 1° vertically, while faces subtended approximately 3.5° horizontally and 5° vertically. The screen then went blank for 200 ms before the fixation cross reappeared and remained on the screen for 250 ms. Each epoch therefore lasted 1250 ms, and each block of trials lasted 80 s. Participants were instructed to maintain fixation at all times. Catch trials occurred pseudo-randomly with an average frequency of one per 16 trials. On a catch trial a word, consonant string or face was followed by a 200 ms blank screen after which the fixation cross re-appeared in green rather than red. Participants were instructed to press a hand-held response button with their left index finger whenever they detected a change to the colour of the fixation cross. Use of the non-dominant left hand was preferred to ensure that any motor-related activation most likely occurred in the non-language dominant hemisphere. In addition, responses to the catch trials were discarded from the data analysis to ensure that the MEG signal was minimally contaminated by motor responses.

### MEG and MRI Data Acquisition

MEG data were collected using a 151 channel CTF Omega system (CTF Systems Inc., Port Coquitlam, Canada) at the Wellcome Trust Laboratory for MEG Studies, Aston University, UK. Data were sampled at 625 Hz with an antialiasing cut-off filter of 200 Hz. In addition, high resolution 1×1×1 mm3 T1-weighted MRI images showing the complete skull were acquired for each participant in sagittal orientation on a Siemens/Varian 3T system fitted with a birdcage head coil (210 sagittal slices; in-plane matrix size 256×256; FOV 256×256×210 mm3; TR 11.2 ms; TE 4.85 ms; TI 300 ms; flip angle 12 degrees). Immediately after finishing MEG data acquisition, a 3-D digitizer (Polhemus Isotrak) was used to digitize the shape of the participant's head in the MEG laboratory and to locate the MEG reference coils for the nasion, left and right ear on the headset, with respect to this head-shape. While the coils were worn in the MEG scanner, small currents were passed allowing them to be localised with respect to the MEG sensor array. Therefore, because the 3-D digitized head shape could be matched to the participant's skull in MRI, so also could the locations of the coils and hence the MEG sensors could be co-registered to the MRI. Artefact rejection was carried out manually, trial by trial, to remove eye blinks and other artefacts.

### Data Analysis

#### Whole Brain Analysis of Word, Consonant String, and Face Responses at 10–20 Hz: A Comparison with Pammer et al [1]

As a first level analysis, we compared the results of the current study with those of Pammer et al. [1]. We therefore used the same parameter settings for beamformer analysis as were employed in that study. This generated statistical parametric maps of stimulus related changes in cortical oscillatory power in the 10–20 Hz frequency band to match Pammer et al., for each of the three experimental conditions.

For each condition, active windows of 200 ms in length were defined. The first active window (0–200 ms) started with the presentation of the stimulus. The active windows were then progressed in 50 ms increments to 300–500 ms. The passive window in each case was the period −200–0 ms, when only the fixation point was present on the screen. Fourier power analysis was used to compare the total amount of power within the 10–20 Hz frequency band between the passive and active windows. The analysis used a volume covering the whole brain in each individual with an interpolated grid size of 5×5×5 mm. A jack-knife statistical method was used to calculate the difference between the spectral power estimates for the active and passive states over all epochs to produce a true t-statistic for each grid point. A 3d *t*-statistic image of differential cortical activity was then generated by repeating this procedure for each grid point in the whole brain. Group-level statistical maps for each condition, for each time window were generated by first registering the individual participant *t* statistic maps to MNI standard space [87], then combining these transformed maps across participants for each time window and frequency band. Registration was performed using FLIRT (FMRIB's Linear Image Registration Tool) [88] to generate the appropriate transforms between each individual's statistical images, their T1-weighted anatomical MRI, and MNI standard space. This transformation matrix was then applied to each of the generated grid points, in each time window, and for each participant. Finally, a simple mixed-effects model was used to generate group statistical *t* maps by combining data across individuals for each contrast.

#### Identification of Virtual Electrode Sites for the Four ROIs: Left IFG, Right IFG, Left MOG, and Left Mid FG (VWFA)

Our objective was to identify four ROIs per participant within which we could compare the timings and amplitudes of the evoked related fields (ERFs) in response to words, consonant strings and faces. In addition we also wanted to investigate the frequency characteristics of these sites as a function of time, to explore the response of the left IFG to words, consonant strings and faces, and to compare the left IFG's response to words with the responses of the right IFG, left MOG and left mid FG (VWFA) to the same stimuli. Therefore, virtual electrode selection needed to be based on beamforming analyses which covered a wider set of frequency bands than those reported in Pammer et al. [1]. To achieve this, we followed the sequence of steps below:

1. Beamformer analyses of individual MEG data (as above), but this time carried out separately for the alpha (8–15 Hz), beta (15–25 Hz) and gamma (25–60 Hz) frequency bands using extended passive and active time windows of −500–0 ms and 0–500 ms respectively.
2. Registration of individual beamformer statistical maps to MNI standard space; combination of maps across participants, separately for each frequency band; generation of group statistics with a simple mixed-effects model, separately for each frequency band.
3. Identifying from the group beamformer statistical maps which frequency band showed the strongest average signal in each of three anatomical areas: bilateral MOG, left IFG, left mid FG.
4. From the selected frequency band, identifying peaks in the group beamformer data corresponding to the four specific sites of interest: left MOG, left IFG, right IFG, left mid FG (VWFA).
5. Matching peaks from the group beamformer data to corresponding peaks at the individual level; extraction of beamformer weights at each peak in each participant and calculation of four time-series per participant.
6. Group analysis of this time-series data in both the amplitude and frequency domains.

For step 3 above, three anatomical masks were created. Two of these masks covered well recognised anatomical regions. The first mask covered the left IFG including the *pars opercularis* and the anterior portion of the precentral gyrus. The second anatomical mask, which was designed to capture occipital responses of the sort seen by Cohen et al. [89], Pammer et al. [1] and others, encompassed visual areas V2, V3 and V4 in BA 18/19 but excluded area V1. A third mask was created to cover the area of the VWFA. That is not a recognised anatomical entity, so a mask was generated that was centred on the mean of the fMRI activation peaks for the VWFA (MNI *X* = −44, *Y* = −58, *Z* = −15; Jobard et al., [25]) with a radius of 5 mm. The three masks were applied to the group statistical data for the words condition in each frequency band, and used to compute the mean *t* score for the ROIs inside the masks. The mean *t* score was highest for the beta frequency band in both the left IFG and VWFA regions, and comparable with the alpha frequency band in the BA18/19 region. We therefore chose the beta frequency band (15–25 Hz) group beamformer maps as the basis for virtual electrode site selection.

Table 1 shows all peaks for the words condition where (*t*>2.5, *p*<0.01) in the group beamforming analysis of the beta frequency band data, using extended time windows (see point 1 above). From this complete list, we then identified the subset of peaks closest to our regions of interest: left MOG, left IFG and left mid FG (VWFA) and these are highlighted in Table 1. To identify the right IFG site, we used the right hemisphere homologue of the left IFG site by taking the positive value of its X co-ordinate. We note that the resulting left mid fusiform peak (MNI *X* = −46, *Y* = −56, *Z* = −18) is remarkably close to the Jobard et al. [25] mean for the VWFA (MNI *X* = −44, *Y* = −58, *Z* = −15). Table 1 also confirms the presence of significant peaks in other parts of the reading network, including the *pars triangularis* region of the left IFG, the left middle temporal gyrus, and the left superior temporal gyrus.
